# Eukaryotic Replicative Helicase Subunit Interaction with DNA and Its Role in DNA Replication

**DOI:** 10.3390/genes8040117

**Published:** 2017-04-06

**Authors:** Matthew P. Martinez, Amanda L. Wacker, Irina Bruck, Daniel L. Kaplan

**Affiliations:** Department of Biomedical Sciences, Florida State University College of Medicine, 1115 W. Call St., Tallahassee, FL 32306, USA; mm14d@my.fsu.edu (M.P.M.); alw16e@my.fsu.edu (A.L.W.); Irina.Bruck@med.fsu.edu (I.B.)

**Keywords:** DNA replication, helicase, protein-DNA interaction, DNA unwinding, DNA melting, replication stress, MCM, Cdc45, GINS

## Abstract

The replicative helicase unwinds parental double-stranded DNA at a replication fork to provide single-stranded DNA templates for the replicative polymerases. In eukaryotes, the replicative helicase is composed of the Cdc45 protein, the heterohexameric ring-shaped Mcm2-7 complex, and the tetrameric GINS complex (CMG). The CMG proteins bind directly to DNA, as demonstrated by experiments with purified proteins. The mechanism and function of these DNA-protein interactions are presently being investigated, and a number of important discoveries relating to how the helicase proteins interact with DNA have been reported recently. While some of the protein-DNA interactions directly relate to the unwinding function of the enzyme complex, other protein-DNA interactions may be important for minichromosome maintenance (MCM) loading, origin melting or replication stress. This review describes our current understanding of how the eukaryotic replicative helicase subunits interact with DNA structures in vitro, and proposed models for the in vivo functions of replicative helicase-DNA interactions are also described.

## 1. Introduction to DNA Replication and Mcm2-7

**Figure 1:**
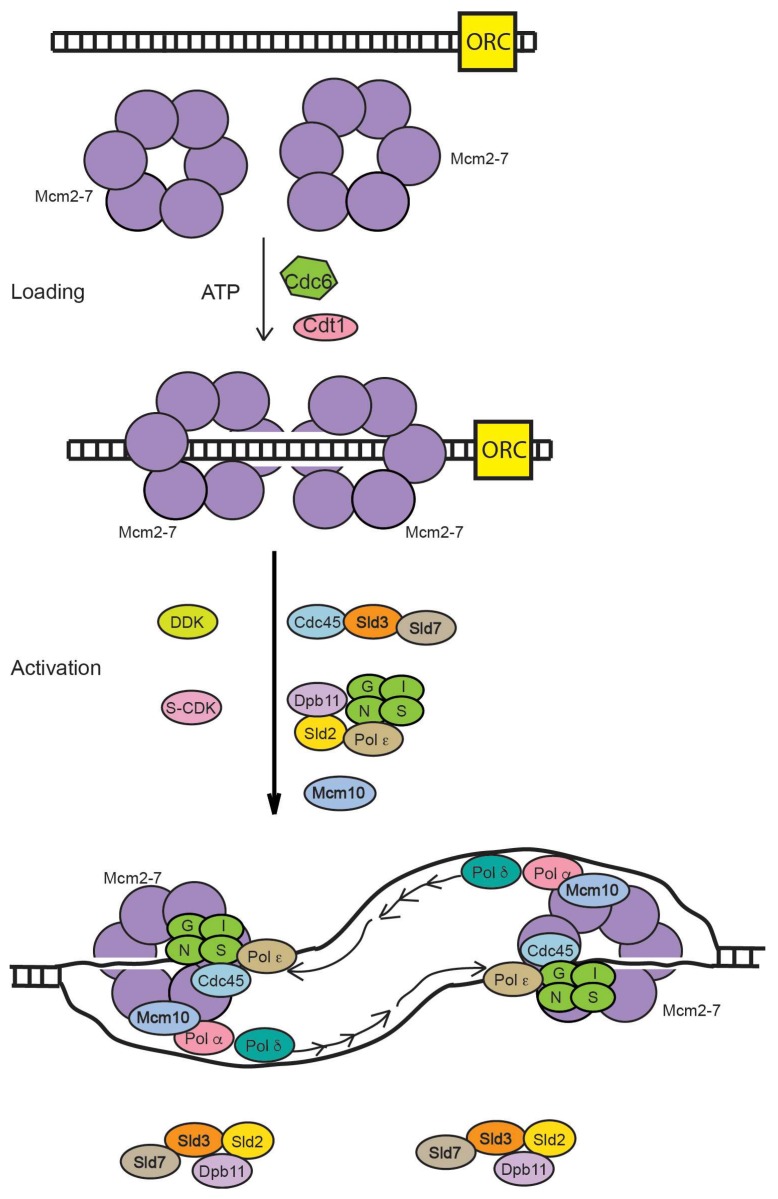
Role of Mcm2-7 in DNA replication. In late M and G1 phases, ORC, Cdc6, and Cdt1 function with ATP to load Mcm2-7 as a double hexameric ring surrounding double-stranded DNA. Mcm2-7 does not unwind DNA in this stage. In S phase, cell cycle kinases (S-CDK and DDK) function with replication initiation proteins (Sld3, Sld2, Dpb11, Sld7, Pol ε, and Mcm10) to assemble Mcm2-7 with Cdc45 and GINS. The CMG complex is the active replicative helicase, unwinding DNA bidirectionally from the origin.

During late M and G_1_ phases of the cell cycle, the Mcm2-7 complex is loaded as a double-hexamer to encircle double-stranded DNA at an origin (Figure 1). The origin recognition complex (ORC), Cdc6, Cdt1, and ATP are required for this activity, which is also known as origin licensing. This reaction has been reconstituted with purified proteins, and recent biochemical and biophysical experiments reveal the detailed mechanism for Mcm2-7 loading [1]. During S phase, the S-phase cell cycle kinases, S-CDK (S-phase cyclin-dependent kinase) and DDK (Dbf4-dpendent kinase), function with the replication initiation factors, including Sld2, Sld3, Dpb11, Mcm10, Pol ε, and Sld7, to assemble Mcm2-7 with Cdc45 and GINS. The Cdc45-Mcm2-7-GINS complex (CMG), fully assembled in S phase, is the replicative helicase in eukaryotes [2]. The CMG helicase unwinds DNA bidirectionally from a replication origin, the site of replication initiation.

The motor region of the eukaryotic replicative helicase is comprised of the heterohexameric Mcm2-7 protein complex. Furthermore, Mcm2-7 alone has some weak in vitro helicase activity under certain conditions [3], but fully active helicase activity requires Mcm2-7 assembly with Cdc45 and GINS [4,5]. By analyzing the crystal structure of MCM (minichromosome maintenance complex) by itself and in complex with nucleotides, DNA, and as the CMG complex, mechanisms of origin melting and DNA unwinding by the Mcm2-7 complex can be further understood. With its role in origin melting and DNA unwinding becoming clearer, more advanced models for its mechanism of unwinding can be developed. Here, the structure of Mcm2-7 and its implications in origin melting are reviewed based on models from archaeal, *Saccharomyces cerevisiae*, *Drosophila melanogaster*, and human systems. Crystallography, FRET (fluorescence resonance energy transfer), structure function, and in vivo experiments have provided much of what is known about the structure and mechanism of this heterohexameric (composed of six different subunits) protein complex.

## 2. DNA Binding Structures of Mcm2-7

**Figure 2:**
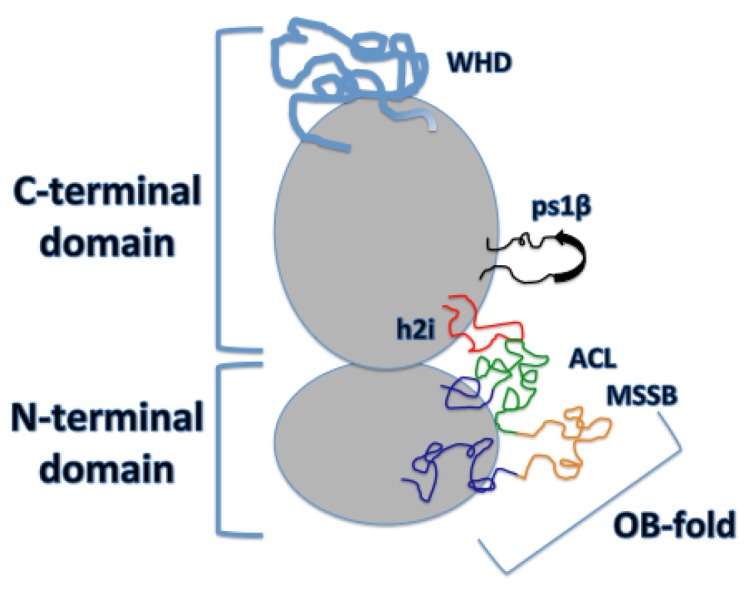
One individual MCM (minichromosome maintenance complex) subunit. Primary DNA-interacting features are shown. The winged helix domain (WHD) in blue; the pre-sensor-1-β hairpin (ps1β) in black; the helix-2-insert (h2i) in red; the OB-fold of the C subdomain of the N-terminal domain (NTD), with the allosteric communication loop (ACL) in green and the MCM single-stranded DNA binding motif (MSSB) in orange. The Mcm2 subunit of *Saccharomyces cerevisiae* does not contain a winged helix domain [11].

Unlike previously studied homohexameric replicative helicases such as papilloma virus E1, SV40 Large T-antigen, archaeal MCM, and *Escherichia coli* DnaB [6], the eukaryotic Mcm2-7 helicase forms a heterohexameric ring structure. The Mcm2-7 hexamer forms a double hexamer when loaded onto origin DNA [7,8]. Mcm2-7 is a two-tiered ring comprised of the N-terminal domain (NTD), required for double hexamer attachment, and a AAA+ C-terminal domain (CTD) that provides the ATPase activity of the helicase (Figure 2) (reviewed in [9]). Both the C-terminal and N-terminal domains of the ring contain DNA binding regions that aid in DNA unwinding and are necessary cell viability [10,11,12].

While the N-terminal domain does not provide any ATPase activity, it is composed of three subdomains that are necessary for DNA binding, double hexamer formation, and helicase activation (reviewed in [2]): a largely helical subdomain A, Zn-binding motif subdomain B, and an OB (oligonucleotide/oligosaccharide-binding)-fold subdomain C. Within the OB-fold, Froelich et al. have identified a region in archaeal MCM where two highly conserved arginine residues, R124 and R186, project from the β-barrel towards the interior of the MCM ring. They termed this region the MCM single-stranded DNA binding motif (MSSB) (Figure 2). They have demonstrated that these residues of the MSSB are necessary for helicase loading and activation [13]. Additionally, they have identified these conserved residues in yeast (as either arginine or lysine) in Mcm4, Mcm6, and Mcm7, and have revealed how alanine mutations in these residues in at least two of the three MCM subunits show significantly reduced helicase loading. Further investigation of the N-terminal domain crystal structure has identified a conserved loop in the OB-fold called the Allosteric Communication Loop (ACL). It has been suggested that the ACL plays a role in N- and C-terminal domain communication, with the ACL positioned near a conserved loop of the AAA+ domain, the pre-sensor-1-β-hairpin (ps1β), of the adjacent subunit. The ACL has also been shown to contact the main-chain amide atoms of the helix-2-inset (h2i) within the same subunit (Figure 2) [14]. This interaction was observed in an inactive MCM, revealing a probable function prior to and during DNA unwinding, discussed later on. In fact, the ACL has an observed influence on unwinding, with several mutants located within this loop showing defects in DNA unwinding [15]. On the other hand, deletion of a fully conserved glutamine residue, identified and characterized by Miller et al., has been shown to increase ATPase activity in archaeal MCM [16]. This implies a role of the ACL in DNA unwinding inhibition; however, further research is needed to completely understand its purpose. While it hasn’t been shown to directly interact with DNA, it interacts with key components of the AAA+ domain and its possible mechanism will be discussed in greater detail later on.

The C-terminal domain of the Mcm2-7 hexamer is the AAA+ domain that provides all of the ATPase activity in the eukaryotic replicative helicase. The active ATPase site, as discussed by Miller et al. [14], consists of Walker-A/B residues of one subunit and three positively charged residues of the adjacent subunit, consisting of sensor-2, the arginine finger, and residues classified as sensor-3. Several modules are directed into the central channel of the CTD that are believed to play an important role in Mcm2-7-DNA interaction. The pre-sensor-1-β-hairpin projects a universally conserved lysine, K785 in archaeal MCM, which is essential for DNA unwinding [17], and the helix-2-insert, required for helicase activity in archaeal MCM, directs family-specific conserved residues R734 and W741 into the central channel (Figure 2) [18]. The h2i projects itself further into the central channel than ps1β and seems to divide the AAA+ and NTD DNA-binding regions [14]. Additionally, five of the six MCM subunits in *S. cerevisiae* MCM contain C-terminal winged helix domains (WHD), with the exception of Mcm2 (Figure 2). In the inactive Mcm2-7 double hexamer, these winged helix domains extend outward from the CTD and appear flexible, yet in the *S. cerevisiae* active CMG complex, the WHD of Mcm5 has been observed to obstruct the interior axial channel of the AAA+ domain with the WHD of Mcm6 stacked on top of it in an active CMG complex [19]. The WHDs of Mcm5 and Mcm6 provide implications for a role in the mechanism of DNA unwinding, discussed later on.

## 3. Varying Mcm2-7 Conformations Leading to CMG Activation

**Figure 3:**
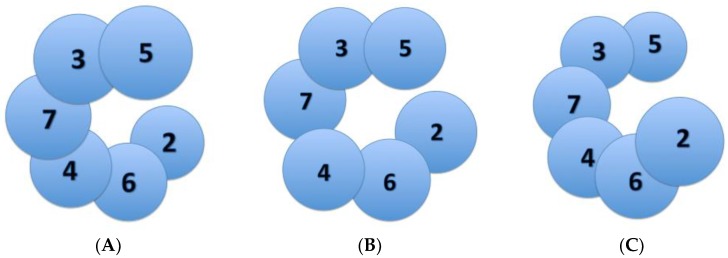
Viewing the Mcm2-7 ATPase domains from under the C-terminal domain. (**A**) a visual representation of the left-handed spiral configuration adopted by Mcm2-7 in its bare state [12,13]; (**B**) a visual representation of the Mcm2-7 hexamer in its relatively planar configuration while present as an inactive Mcm2-7 double hexamer [3,13]; (**C**) the observed right-handed configuration of Mcm2-7 in its active CMG, DNA-bound state [14].

**Figure 4:**
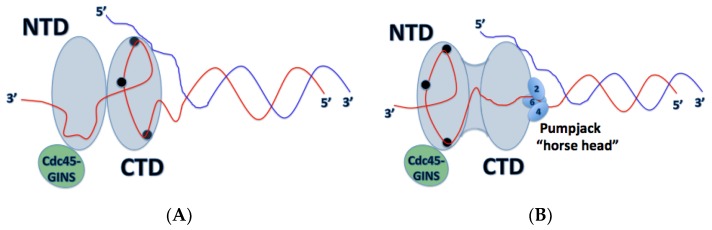
A view of the CMG helicase from a cut-away side view in its proposed pumpjack mechanism of Mcm2-7 translocation along ssDNA. The red strand indicates the leading strand DNA. (**A**) the Mcm2-7 hexamer in its compact state, where the CTD is bound to ssDNA. ssDNA passes through each domain 5′ → 3′ in a clockwise fashion. The black circles in the CTD represent the h2i/ps1β motifs, bound to ssDNA; (**B**) the Mcm2-7 helicase is in its relaxed configuration with the “horse head” (consisting of subunits 2, 4 and 6) of the pumpjack up. DNA is not entirely bound to the CTD; however, it remains bound to the OB-folds (black circles) of the NTD.

The eukaryotic Mcm2-7 heterohexamer is believed to exhibit many different conformational states that help ensure its regulation in origin licensing, melting, and further DNA unwinding (Figure 3). An earlier study from Costa et al. in metazoan MCM demonstrated that the bare Mcm2-7 complex adopts a notched, left-handed spiral conformation [20]. This finding was later confirmed by the slight left-handed spiral conformation of recombinant human Mcm2-7 in complex with the non-hydrolysable ATP homolog ATPγS (Figure 3A) [21]. Once loaded onto origin DNA, two Mcm2-7 hexamers join together, connected by the Mcm2-7 N-terminal domains, to form a planarized double hexamer (Figure 3B) [8,21]. Upon helicase activation by GINS and Cdc45 to form the active CMG complex, the Mcm2-7 AAA+ ATPase subunits form a right-handed spiral around ssDNA (Figure 3C) [22]. In fact, the N-terminal domain remains relatively planar. The more recent research from Yuan et al. demonstrated how Cdc45-GINS interactions with the Mcm2-7 hexamer actually stabilizes the NTD and allows for flexibility within the AAA+ domain [19]. Interestingly, they point out that the only contacts of Cdc45 with Mcm2 and Mcm5 are with the NTD of the hexamer, making no contacts with the CTD (Figure 4). This provides implications for additional findings by this group and others, showing how the active CMG AAA+ domain fluctuates between planar and tilted forms. Along with Yuan et al., another team of researchers simultaneously identified two similar conformations of the active CMG helicase, where the AAA+ motor exists in two conformations: a compact (tight), planar configuration and a relaxed (notched), right-handed spiral configuration (Figure 4) [19,23].

These discontinuous, spiral configurations are made possible by a natural discontinuity in the Mcm2-7 ring at the Mcm2-5 interface (Figure 3). This Mcm2-5 gate is believed to play a role in the conversion from dsDNA to ssDNA in the central channel of this helicase, and Mcm2-5 gate opening may be stimulated by DDK phosphorylation of Mcm2 [24]. In the relaxed, elongated Mcm2-7 configuration, Cdc45-GINS is wedged between the N- and AAA+ domains, where there is about an 8.2 ‎Å difference in the distance between Mcm2 and Mcm5 between the open and closed configurations [23]. Movement of the CMG between these two conformations depends primarily on the Mcm2-5 interface, with its loosening chaperoned by compensatory tightening of the neighboring Mcm3-5 and Mcm2-6 interfaces. This tightening and loosening of the Mcm2-5 interface to facilitate movement between a planar, tight configuration (Figure 3B) and a right-handed spiral, notched configuration (Figure 3C) may provide insights into the mechanism by which the CMG melts origin DNA and translocates along the ssDNA.

## 4. Mechanism of DNA Unwinding and Translocation by the Mcm2-7 Helicase

**Figure 5:**
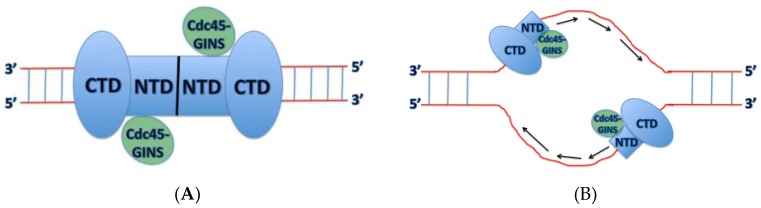
The newly proposed DNA polarity and direction of CMG translocation. (**A**) the two active CMG complexes bound by the Mcm2-7 N-terminal domains on dsDNA prior to origin melting; (**B**) the two active CMG complexes translocate in a 3′ → 5′ direction with the N-terminal domain in front of the C-terminal domain. The two CMG complexes must encircle ssDNA before they can pass each other and unwind further duplex DNA.

Inactive Mcm2-7 hexamers are loaded onto dsDNA where they form inactive Mcm2-7 double hexamers, bound by the Mcm2-7 N-terminal domains. How the origin dsDNA is melted into ssDNA and one strand is excluded from the central channel of the Mcm2-7 ring still remains unclear; however, recent structural and biochemical data have suggested some possible mechanisms for action. One interesting finding by Costa et al. revealed that in the dimeric CMG state prior to separation, Cdc45-GINS (and therefore the Mcm2-5 gate) are oriented towards opposite sides of the rings, offset by 180° (Figure 5) [22]. This could provide some insight into the mechanism of origin melting, since this configuration would allow a strand of DNA to escape from the Mcm2-7 ring without steric interference from the partner CMG. Interaction between single-stranded origin DNA and replication initiation factors, including Dpb11, Sld3, Sld2, and Mcm10, may also function to promote origin melting [25,26,27,28].

In the *D. melanogaster* model, dsDNA is shown to enter the C-terminal pore, where one of the single DNA strands contacts ps1β and h2i loops of three MCM subunits, 7-4-6, in a 5’ → 3’ clockwise fashion [23]. A previous finding can support this, where ssDNA binds with the same polarity to the MSSB in archaeal MCM [13]. These findings contribute to a suggested model of DNA unwinding and translocation by Miller et al., where in the compact ATPase site, the h2i and ps1β are expected to move upwards, binding to a strand of DNA and increasing the distance between the ACL and the ps1β. Upon ATP hydrolysis and transition into the relaxed state [23], the h2i/ps1β would be driven downward to translocate one ssDNA strand while the other strand is excluded from the ring [14]. Together, these findings support the pumpjack motion of CMG translocation on a single strand of DNA, proposed by Yuan et al., where the C-terminal domains of the CMG complex ratchet up and down like a pumpjack and move the helicase along ssDNA like an inchworm (Figure 4) [19]. In this model of translocation, one can see a ratcheting type of expansion/contraction movement, between the notched and compact configurations of Mcm2-7. The NTD-Cdc45-GINS subcomplex acts as a rigid platform for which the CTD can flex and the Mcm2-Mcm6-Mcm4 act like the nodding horse head. In the ATP bound form, CMG is in its compact configuration causing the Mcm2-6-4 head to nod (Figure 4A). Upon ATP hydrolysis, the Mcm2-5 interface springs open causing subsequent upward movement of the Mcm2-6-4 head (Figure 4B). Based on findings from Ali et al. and this pumpjack motion of translocation, the DNA strand is bound by the AAA+ domain when the Mcm2-6-4 head is nodded and the hexamer is in its compact configuration. When the ATPases are moved upwards and the hexamer is in its relaxed configuration, the DNA strand is passed on to NTD OB-fold MSSB (Figure 4B) [23]. Interestingly, with the Mcm5 WHD protruding into the central channel of the CTD ring, strand separation may be facilitated here since the WHD restricts the diameter of the central pore to ~10 Å, a diameter too small to accommodate dsDNA [19]. Viewed from above the C-terminal domain of Mcm2-7 in complex with DNA, the winged helix domain of Mcm5 obstructs the central pore of the helicase, restricting the diameter to a width that can only accommodate ssDNA and not dsDNA. This possibility is supported by previous findings that the CMG helicase moves 3′ → 5′ along the leading strand, with the C-terminal domain leading the N-terminal domain in translocation [13,17,22]. These findings together support a steric exclusion mechanism of duplex DNA unwinding [19,23].

A very recent publication has challenged a commonly accepted idea about the mechanism of action undergone by the CMG helicase in DNA translocation. In this new research, the team investigated an active CMG complex from *S. cerevisiae* in complex with a forked DNA substrate, obtaining some of the highest resolution images reported so far. In this model, the team of researchers discovered that DNA traverses through the Mcm2-7 heterohexamer 5′ → 3′ from the N-terminal domain to the C-terminal domain (Figure 5) [29]. This goes against the DNA polarity in relation to the Mcm2-7 helicase previously reported [22]. The previous study by Costa et al. examined *D. melanogaster* CMG, so it is possible that the two have opposite orientations on DNA. While this is the first finding of its kind regarding the CMG helicase, it is possible that there is some conservation for this mechanism and that it may exist in other eukaryotic species. Previous studies of papillomavirus E1 have displayed a similar mechanism, where the N-terminal domain (DBD in E1) leads the ATPase domain (HD in E1) and the two hexameric helicases must pass each other upon DNA melting and translocation, similar to the mechanism proposed in Georgescu et al. [30,31]. Further studies of CMG in complex with forked-DNA will need to be conducted to investigate these discrepancies. This opens the door to more questions; how does the CMG helicase unwind DNA? What does this reveal about Cdc45-DNA and GINS-DNA mechanisms? Is the mechanism of DNA unwinding by CMG conserved among eukaryotes? It was shown that the leading strand is threaded through the NTD, while the lagging strand is possibly captured by the NTD-bound pol α-primase, potentially reducing the amount of exposed ssDNA. It isn’t clear, however, how the exact mechanism of how this unwinding occurs.

The new proposed polarity of the Mcm2-7 complex with respect to the parental duplex DNA also raises new questions regarding the interaction of the polymerases and accessory subunits with the replisome. Are the three replicative polymerases, pol α, pol δ, and pol ε, positioned in front of the CMG helicase, behind the CMG helicase, or at the side of the CMG? What is the architecture of Mcm10 and Ctf4 proteins that connect the helicase to the polymerases, relative to the replication fork? Future structural studies in this field will help resolve this issue, but studies by the O’Donnell lab suggest that leading strand Pol ε is positioned on the opposite side of the helicase relative to Ctf4 and the lagging-strand polymerase (Pol) α-primase [32]. Furthermore, a recent biochemical study indicates that Mcm10 stimulates the helicase activity of CMG [33], suggesting that Mcm10 may play a key role in dissociating the twin Mcm2-7 hexamers during replication initiation [33,34,35].

Georgescu et al. have also demonstrated that Mcm3, Mcm5, Mcm2, and Mcm6 of the CTD interact with ssDNA [29]. This, combined with previous reports that Mcm4, Mcm6, and Mcm7 contact ssDNA [23], indicates that all of the subunits play a role in ssDNA binding. The findings from Georgescu et al. indicate that each subunit binds 2 nucleotides of DNA. These new findings provide new possibilities of the mechanism or origin melting in eukaryotes. The CTD of the Mcm2-7 ring pushes the NTD forward, and before activation of the CMG complex, two Mcm2-7 hexamers are head-to-head, joined by the Mcm2-7 N-terminal domains forming a double hexamer (Figure 5A). Therefore, this may provide a newly recognized quality control mechanism at the origin, as described in [29]. The authors herein describe how with this head-to-head, N-terminus to N-terminus interaction, each hexamer must encircle ssDNA before the N-terminal rings can move forward, with the pushing action of the AAA+ domain, and pass each other (Figure 5B). Additionally, this head-on orientation of each CMG hexamer may provide the energy required for origin melting. The mechanism for initial dsDNA melting remains unknown, and further research is needed to investigate this possibility.

Recent work has also begun to elucidate the mechanism for replication termination. During replication termination, the CMG is released from chromosomal DNA. This activity depends upon ubiquitylation of its Mcm7 subunit by a Cullin–RING E3 ubiquitin ligase [36,37]. Interestingly, the modification of Mcm7 recruits the p97/VCP segregase, an AAA-ATPase that dismantles protein complexes in a ubiquitin-dependent manner [38,39]. Note that is not the typical protein degradation that is characteristic of ubiquitin-dependent pathways.

## 5. Cdc45 Architecture

Cdc45 is required for DNA replication, and Cdc45 is a key protein in the CMG replicative helicase in eukaryotes [40]. Initially, no homolog of Cdc45 was detected in bacteria or archaea. However, thorough bioinformatic analysis revealed in June of 2011 that Cdc45 is homologous to the bacterial RecJ protein, a 5′- to 3′-single-stranded DNA exonuclease [41]. RecJ has multiple roles in DNA repair, and RecJ is a member of the DHH phosphoesterase superfamily (the DHH is named for an amino acid motif, aspartate-histidine-histidine, that is commonly found in the DHH phosphoesterase superfamily).

Later that year, a biochemical and structural analysis of Cdc45 revealed that Cdc45 has a protein architecture similar to RecJ, confirming that the two proteins are related to one another [42]. The protein architecture of human Cdc45 was determined by small angle X-ray scattering in this manuscript. The authors also demonstrated for the first time that Cdc45 binds to single-stranded DNA, but not double-stranded DNA [42]. Interestingly, the authors also showed that, unlike RecJ, Cdc45 does not possess nuclease activity [42].

In 2016, the Pelligrini lab determined the crystal structure of human Cdc45 at 2.1 angstroms resolution [43]. The crystal structure confirmed the evolutionary link with the bacterial RecJ exonucleases. However, the catalytic residues of RecJ exonuclease are missing in the Cdc45 sequence. The structure revealed that Cdc45 adopts a compact, disk-like shape, resulting from folding of the protein into a closed arc. The DHH domain of Cdc45, encompassing residues 1 through 229, is a five-stranded parallel beta-sheet surrounded by three alpha helices on either side. The DHHA1 domain of Cdc45, which is usually present in DHH phosphoesterases, encompasses residues 439 to 566 and consists of four-stranded beta-sheets flanked by one and two helices on either side. The sequence in the central region of Cdc45, spanning the amino acids between the DHH and DHHA1 domains, does not belong to the RecJ family, and is unique to Cdc45. This region mediates the interaction between Cdc45 and Mcm2-7, and between Cdc45 and GINS.

## 6. Cdc45 Interaction with DNA

The role of Cdc45 binding to single-stranded DNA has been explored by different research groups. First, work from our lab found that the interaction between Cdc45 and 60-mer single-stranded DNA is important during replication stress, since we found that Cdc45 mutants defective for single-stranded DNA interaction were sensitive to hydroxyurea [44]. Hydroxyurea depletes nucleotide pools and causes the polymerases to stall behind the replicative helicase. We also found that Cdc45 interaction with single-stranded DNA weakened the interaction between Cdc45 and Mcm2-7, suggesting that Cdc45 interaction with 60-mer single-stranded DNA may stall the replicative helicase [44]. We proposed that Cdc45 binding to 60-mer single-stranded DNA might occur when the polymerases stall during replication stress, and that Cdc45 interaction with single-stranded DNA is a mechanism to coordinate polymerase stalling with helicase stalling [44].

Work from Grosse, Popsiech and colleagues suggest that Cdc45 binds to the 3′-protruding strands of single-strand/double-strand DNA junctions [45]. Furthermore, these investigators found that Cdc45 may slide along single-stranded DNA in the 3′- to 5′-direction [45]. The authors propose that Cdc45 may act as a wedge to drive strand displacement at a replication fork [45]. Recently, work from this group of investigators found that Cdc45 can help deposit RPA (replication protein A), the eukaryotic single-stranded binding protein, onto single-stranded DNA, suggesting an additional function for Cdc45 interaction with single-stranded DNA [46]. The authors suggest that Cdc45 may help load RPA onto newly emerging ssDNA at an ongoing replication fork (the replication fork is the site where the parental DNA duplex is split by the replicative helicase) [46].

Botchan, Berger and colleagues also investigated the function of Cdc45 interaction with single-stranded DNA [47]. The authors first discuss that Mcm2-7 can exist in equilibrium between a closed-ring and opened-ring state [20]. In the opened-ring state, there is discontinuity between the Mcm2 and Mcm5 subunits [48]. The authors find that in the opened-ring state, the leading strand of a model replication fork binds specifically to Cdc45 [47]. Furthermore, the authors show that mutations in Cdc45 that abolish this interaction diminish helicase activity [47]. The authors propose a model in which Cdc45 serves as a shield to guard against the occasional slippage of the leading strand from the central channel of the CMG [47].

## 7. GINS Architecture

**Figure 6:**
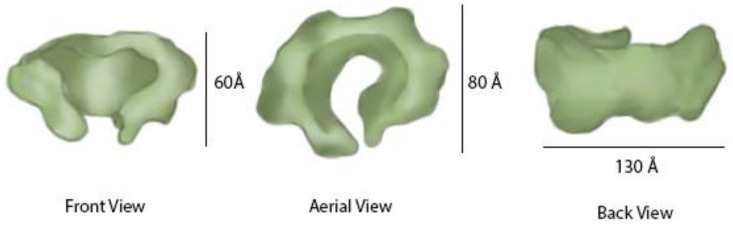
Architecture of the GINS complex. The architecture of the GINS structure, determined in isolation, is shown.

GINS is a macromolecule protein complex, whose name is derived from its four subunits [49]. The first letter of the Japanese number associated with each protein is used the initial of each subunit. Sld5 (“Go”), Psf1 (“Ichi”), Psf2 (“Ni”), and Psf3 (“San”) form the heterotetrameric GINS complex with a 1:1:1:1 stoichiometric ratio. In an analysis conducted by Jasminka Boskovic et al., the GINS complex was determined to be shaped like a “C” or “horseshoe” (Figure 6) [50]. There are no connections between Sld5 and Psf3 and no connections between Psf1 and Psf2.

The GINS complex is arranged somewhat like a funnel, with the upper parts of the complex having a wider opening in the center measuring about 70 Å that becomes narrower, leading to a central hole or pore measuring about 30 Å (Figure 6). The pore is partially regulated by the Psf3 N-terminal residues that fit loosely into the pore [51].

**Figure 7:**
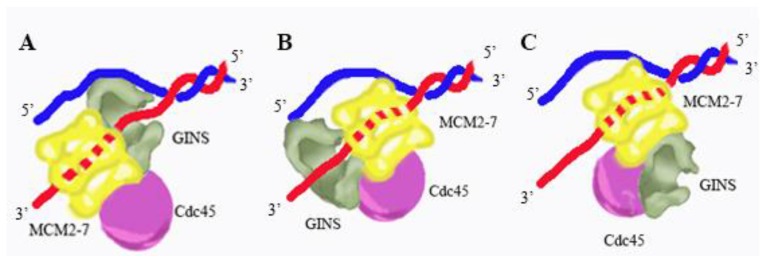
Model for CMG replicative helicase architecture at a fork. Three different models for CMG replicative helicase architecture at a fork are shown. In each of these models, CMG unwinds DNA by a steric exclusion mechanism. (**A**) in model A, GINS is positioned at the front of the fork, gripping single-stranded DNA and presenting the ssDNA to the Mcm2-7 ATPase; (**B**) in model B, GINS is positioned at the back of the fork, gripping leading strand DNA as it exits from the Mcm2-7 complex; and (**C**) in model C, GINS is at the side of the Mcm2-7 complex and GINS does not bind fork DNA under normal conditions. Recent electron microscopy studies suggest that Model C is relevant for a replication fork under normal conditions.

Y. Paul Chang et al. crystalized the full-length human GINS complex in order to further determine the functional role of the complex (Figure 7) [51]. Each subunit’s structure is comprised of a domain composed of α-helical bundles. Each unit has a smaller domain, which contains a three-stranded β-sheet core. The GINS subunits connect to each other with the sides of the α-helical domains into a ring like structure starting with Sld5 connecting with Psf1, Psf1 connecting to Psf3, Psf3 connecting to Psf2, and Psf2 connecting back to Psf5, closing the horseshoe shape slightly. The subunits are paralogs with close relationships between Psf1 and Sld5, and also between Psf2 and Psf3.

Interactions create extensive contact between the adjacent subunits, providing a large interface area. This large interface area creates strong bonding forces, which makes the entire tetrameric GINS complex highly stable, even in solution. Electron microscope analysis conducted by Y. Paul Chang et al. showed regions on the surface of the proteins Sld5, Psf1 and Psf3 that showed no electron density in the crystalized structures [51]. Chang et al. attribute the lack of density to the regions’ flexibility. The location of the regions on the surface area facing outwards suggests they might be binding sites for the partner proteins Mcm2-7 and Cdc45 [51].

At the cell’s transition from G_1_ phase to S phase, GINS is recruited to the DNA origin replication site [52]. During replication initiation, GINS binds to Cdc45 and Mcm2-7 to create the CMG (Cdc45/Mcm2-7/GINS) complex, considered to be the replicative helicase [4]. GINS has been identified to be a factor essential for the initiation of DNA replication, as well as normal progression of the replisome [53,54]. 

The crystal structure of GINS differs from the electron microscopy structure of GINS when GINS is part of the CMG. Recent biophysical data demonstrates that GINS can exist as a compact tetramer or also as a double-tetramer in solution [55]. The double-tetramer of GINS that exists in solution may represent an intermediate that occurs prior to CMG helicase separation [55].

## 8. GINS Interaction with DNA

J. Boskovic et al. conducted an experiment that involved binding purified human GINS complexes to DNA probes made to resemble various replicative structures that were then analysed by the Electrophoretic Mobility Shift Assay (EMSA) [21]. The DNA probes were made to resemble single stranded DNA (ssDNA), double stranded DNA (dsDNA) and a “bubble-shaped” DNA [50]. GINS was found to prefer probes of ssDNA or probes containing stretched of ssDNA compared to probes made up of dsDNA [50]. A “super shift” was observed in the “bubble” probe. The slower migrating species observed for the “bubble” probe could be the result of GINS bound to either of the ssDNA strands of the “bubble” [50]. Although the GINS functional role within the CMG complex is unclear, these findings suggest that GINS can directly associate with DNA. 

There are three possible models for GINS interaction with DNA in the context of the moving CMG. In the first model, GINS is ahead of the Mcm2-7 complex, delivering single-stranded DNA to the Mcm2-7 ring (Figure 7A). This model is consistent with the binding preference of GINS for branched DNA structures [21,50]. In the second model, GINS is at the back of the fork, delivering single-stranded DNA to the replicative polymerases (Figure 7B). This is consistent with the ability of GINS to bind single-stranded DNA [21,50]. In the third model, GINS does not bind DNA at all during normal DNA replication. This model is consistent with recent electron microscopy structures of the CMG complex, which do not show direct interaction between GINS and DNA [19,22,29,43]. It may be that GINS plays a role in binding DNA during replication stress or some other condition. For example, GINS has been shown to dissociate from Cdc45 and Mcm2-7 during replication fork collapse [56]. A key experiment in the future will be to identify residues of GINS required for DNA interaction, and then use site-directed mutagenesis to determine the role of GINS-DNA interaction in the cell.

## 9. Conclusions

The CMG unwinds DNA at a replication fork in the nucleus of eukaryotic cells, providing single-stranded DNA templates for the replicative polymerases. Interaction between Mcm2-7 and single-stranded DNA is required for powered-motion of the helicase along the leading DNA strand [17]. ATP fuels the Mcm2-7 complex as it transits along leading strand DNA [57]. CMG translocation along leading strand DNA accomplishes replication fork unwinding by a steric exclusion mechanism [58]. Thus, the interaction between Mcm2-7 and leading strand DNA is essential for DNA replication progression. 

During the initiation of DNA replication, the origin DNA is converted from double-stranded DNA to single-stranded DNA, in a process known as origin melting [9]. The process of origin melting is essential for replication initiation and activation of the helicase, since the CMG unwinds DNA by a steric exclusion mechanism [58]. While the mechanism for origin melting is currently not known, Mcm2-7, Sld2, Sld3, Dpb11, and Mcm10 interaction with DNA may play some role in the initial melting of the DNA duplex [9,13]. Cdc45 interaction with single-stranded DNA may be required during times of replication stress [44], as the Cdc45 protein is uniquely positioned within the CMG architecture to guard against DNA slippage from the helicase [47]. GINS also binds to DNA in vitro [21], but the in vivo role for GINS-DNA interaction has not yet been elucidated. Future directions include determining how origin DNA is melted during replication initiation, and further characterizing in molecular detail how the CMG unwinds DNA during replication fork progression.
